# Predictors of Prolonged Mechanical Ventilation after Open Heart Surgery

**DOI:** 10.15171/jcvtr.2014.014

**Published:** 2014-12-30

**Authors:** Ziae Totonchi, Farah Baazm, Mitra Chitsazan, Somayeh Seifi, Mandana Chitsazan

**Affiliations:** ^1^Department of Cardiac Anesthesiology, Rajaei Cardiovascular Medical and Research Center, Iran University of Medical Sciences, Tehran, Iran; ^2^Rajaei Cardiovascular Medical and Research Center, Iran University of Medical Sciences, Tehran, Iran; ^3^Shahid Beheshti University of Medical Sciences, Tehran, Iran

**Keywords:** Airway Extubation, Cardiopulmonary Bypass, Coronary Artery Bypass, Ventilator Weaning

## Abstract

***Introduction:*** Due to the importance of prolonged mechanical ventilation (PMV) as a postoperative complication, predicting "high-risk" patients by identifying predisposing risk factors is of important issue. The present study was aimed to identify perioperative variables associated with PMV in patients undergoing open heart surgery.

***Methods:*** A total of 743 consecutive patients, American Society of Anesthesiologists (ASA) physical status class III, who were scheduled to undergo open heart surgery using cardiopulmonary bypass were included in this observational study. Perioperative variables were compared between the patients with and without PMV, as defined by an extubation time of >48 h.

***Results:*** PMV occurred in 45 (6.1%) patients. On univariate analysis, pre-operative variables; including gender, history of chronic obstructive pulmonary disease (COPD); chronic kidney disease and endocarditis, intra-operative variables; including type of surgery, operation time, pump time, transfusion in operating room and postoperative variables; including bleeding and inotrope-dependency were significantly different between patients with and without PMV (all P<0.001, except for COPD and transfusion in operating room; P=0.004 and P=0.017, respectively).

***Conclusion:*** Our findings reinforce that risk stratification for predicting delayed extubation should be an important aspect of preoperative clinical evaluation in all anesthesiology settings.

## Introduction

Prolonged mechanical ventilation (PMV), also known as delayed extubation, is an important complication following cardiovascular surgeries. Although occurs in only 3-9.9% of patients, it may be associated with considerable morbidity and mortality.^1-7^ A significant number of these patients will undergo tracheostomy.^8^ Patients who experience delayed extubation will have longer intensive care unit (ICU) and hospital length of stay, higher treatment cost and lower quality of life.^9-14^ However, there is no general consensus regarding the exact definition of delayed extubation. Previous reports have used several definitions ranging from extubation time of greater than 24 h to greater than seven days.^12,14-17^

It is believed that delayed extubation occurs in “high-risk” patients who can be easily identified preoperatively or upon arrival in the ICU after operation. In the previous literature attempts have been made to recognize these risk factors and their reliability to predict delayed extubation following cardiac surgery.

However, still data are lacking regarding these risk factors in Iranian adult patients undergoing open heart surgery using cardiopulmonary bypass (CPB). Accordingly, in the present study we aimed to elucidate the patient characteristics and perioperative variables that predict PMV in Iranian adult population undergoing open heart surgery using CPB.

## Materials and methods

After approval by the Research Ethics Committee of Rajaei Cardiovascular, Medical and Research Center and after obtaining written informed consent from all patients, 743 consecutive adult patients >18 years of age, American Society of Anesthesiologists (ASA) physical status class II or III, who were scheduled to undergo open heart surgery using CPB were included in this prospective observational study.

Premedication included oral lorazepam (1 mg) the night before surgery and intramuscular morphine (0.1 mg/kg) and oral lorazepam (1 mg) approximately 1 h before surgery for all patients. Anesthesia was induced by midazolam (0.1 mg/kg), sufentanil (0.1-0.3 µg/kg) and atracurium (0.5 mg/kg). Anesthesia was maintained using midazolam (1 µg/kg/min), sufentanil (0.005-0.01 µg/kg/min) and atracurium (6 µg/kg/min). Patients underwent median sternotomy. Myocardial protection was achieved with cold potassium-rich ringer solution as the cardioplegic agent. All patients underwent standard bypass with oxygenation and moderate hypothermia. Patients were actively rewarmed to 38 °C before removal of the aortic cross-clamp and weaning from CPB.

Preoperative, intraoperative and postoperative variables were collected. Preoperative collected variables included age; sex; body mass index (BMI); left ventricular ejection fraction (LVEF); hemoglobin level; any medical history of hypertension, diabetes, chronic obstructive pulmonary disease, hypothyroidism, chronic renal failure, and endocarditis; previous use of corticosteroids; history of smoking and opioid addiction. Type of surgery [valvular versus coronary artery bypass grafting (CABG)] and timing of the surgery (emergency versus elective) were also determined.

Intraoperative variables included duration of surgery and CPB and the need to blood transfusion in the operating room. Bleeding during the first 24 h after surgery and the dependency to inotropes (including epinephrine, dobutamine or dopamine) after surgery were the collected postoperative variable. Prolonged mechanical ventilation, defined as extubation time of >48 h, was considered the outcome variable.

Bleeding was defined as either transfusion of ≥5 U whole blood or packed red blood cells within a 48-hour period or reoperation after closure of sternotomy for the purpose of controlling bleeding [also known as Bleeding Academic Research Consortium (BARC)  type 4 (CABG-related) bleeding].^18^

Chronic kidney disease was defined as a glomerular filtration rate (GFR) <60 mL/min/1.73 m^2^ for 3 months or more, according to the recommendations by Kidney Disease Quality Outcome Initiative (K/DOQI).^19^ Hypothyroidism was defined as a thyrotropin (TSH) level of >4.5 mIU/L and/or current levothyroxine therapy. Patients were found to have chronic obstructive pulmonary disease (COPD) if they had clinical evidence of chronic bronchitis, emphysema or small airway disease confirmed by spirometry.

According to the guideline by the Canadian Critical Care Clinical Practice Guidelines Committee^20^, adequate nutritional support was applied both pre- and post-operatively to provide sufficient macro- and/or micronutrient supply for all patients.

Stress ulcer prophylaxis was achieved in all patients by intravenous H2 receptor antagonists or proton pump inhibitors during the operation and ICU stay. Chest physiotherapy was prescribed routinely as soon as possible for all patients post-operatively, even for those who were still mechanically ventilated. Mucolytic agents were used in patients with abnormal or viscid bronchial secretions, at the discretion of the attending pulmonologist. Delirium, if occurred, was treated with adequate doses of benzodiazepines and/or haloperidol.

### Statistical analysis

All analyses were conducted by Statistical Package for Social Sciences (SPSS) software, version 19 (SPSS, Chicago, IL). Data are expressed as mean ± SD or numbers and percentages. All data initially were analyzed using the Kolmogorov-Smirnov test to assess for normality. Quantitative variables were compared using the Chi-squared test or Fisher’s exact test when appropriate. Qualitative variables were compared using the Student’s t-test and Mann-Whitney U test for numerical variables. All *P*-values were two-tailed and *P*<0.05 was considered statistically significant.

## Results

Seven hundred and forty three patients, including 497 (66.89%) male and 246 (33.10%) female were enrolled in the study. The mean age of the study population was 56 ± 14 years. A comprehensive list of preoperative variables is listed in Table 1.

**Table 1:** Results of the univariate analysis of preoperative factors in the early and delayed extubation groups

**Variable**	**Early extubation** **(n=698)**	Delayed extubation* **(n=45)**	***P *** **-value**
Sex (M/F)	479 (69)/219 (31)	18 (40)/27 (60)	<0.001
Age (yr)	56±14	56±13	0.610
BMI (kg/m^2)^	25±4.59	25±4.17	0.675
Hypertension	263 (38)	29 (64)	<0.001
Diabetes	216 (31)	11 (24)	0.359
COPD	28 (4)	6 (13)	0.004
Hypothyroidism	49 (7)	3 (7)	0.928
Chronic kidney disease	71 (10)	17 (38)	<0.001
Endocarditis	1 (<1)	3 (7)	<0.001
History of corticosteroids use	27 (4)	2 (5)	0.847
Smoking	144 (21)	5 (12)	0.122
Opioid use	80 (11)	4 (9)	0.597
Timing of surgery			
Emergency	63 (9)	5 (11)	0.638
Elective	635 (91)	40 (89)	
LVEF			
<30%	133 (19)	7 (16)	0.561
>30%	565 (81)	38 (84)	
Hb			
>10 mg/dl	94 (13)	7 (16)	0.656
<10 mg/dl	604 (87)	38 (84)	

BMI, body mass index; COPD, chronic obstructive pulmonary disease; Hb, hemoglobin; LVEF, left ventricular ejection fraction *Extubation of >48 h after surgery

Among all patients, 45 (6.1%) had prolonged mechanical ventilation. Age and BMI were not significantly different between patients who extubated before and after 48 h post-operation (*P*=0.610 and 0.675, respectively). However, PMV was more prevalent among female patients (*P*<0.001). Significant differences in the prevalence of hypertension, COPD, chronic kidney disease and history of endocarditis were seen between the two groups of patients who extubated either before or after 48 h (*P*<0.001, 0.004, <0.001 and <0.001, respectively). No significant differences were also observed in the low-LVEF (<30%) and low-hemoglobin level (<10 mg/dl) between the two groups (*P*=0.561 and 0.656, respectively). According to the timing of the surgery, although the majority of patients with PMV have been undergone cardiac surgery emergently but this value did not reach statistically significant level (*P*=0.638).

Moreover, significant differences were found between the two groups with respect to the type of surgery and durations of surgery and CPB (all *P*<0.001) (Table 2). Patients who underwent blood transfusion in the operating room were more probable to have delayed extubation time as compared to those patients without transfusion (*P=* 0.017). Prevalence of bleeding during the first 24 h after operation and dependency to inotropes after surgery were also significantly higher in patients with delayed extubation (both *P*< 0.001) (Table 2).

**Table 2:** Results of the univariate analysis of perioperative factors in the early and delayed extubation groups.

**Variable**	**Early extubation** **(n=698)**	Delayed extubation* **(n=45)**	***P*** **-value**
Type of surgery			
CABG	421 (60)	14 (31)	<0.001
Valvular	173 (25)	24 (53)	
Others	104 (15)	7 (16)	
Operation time			
<4 h	431 (62)	3 (7)	<0.001
>4 h	267 (38)	42 (93)	
Pump time			
<60 min	114 (16)	2 (4)	<0.001
60-120 min	400 (57)	19 (43)	
>120 min	184 (27)	24 (53)	
Transfusion in OR	418 (60)	35 (78)	0.017
Bleeding**	14 (2)	31 (69)	<0.001
Inotrope dependency	146 (21)	33 (73)	<0.001

CABG, coronary artery bypass grafting; OR, operating room. Date are represented as numbers (%). *Extubation of >48 h after surgery **Type 4 or CABG-related BARC bleeding

## Discussion

Early weaning of patients from mechanical ventilation after cardiac surgery enhances the cardiopulmonary function and early ambulation, reduces the length of ICU and/or hospital stay, and causes an improvement in the intrapulmonary shunt fraction after extubation.^21^

Although the predictors of delayed extubation cannot be defined easily, the ability to identify high-risk patients and pre- and perioperative risk factors may help to develop surgical and medical modifications which will allow earlier extubation.

There has been a great deal of interest concerning predictive indicators of delayed extubation in patients undergoing cardiac surgeries in the recent decade. Wong et al^22^ in a prospective study on 885 patients undergoing CABG showed that advanced age and female gender increase the risk of delayed extubation.They also believed that intra-aortic balloon pump, inotropes, excessive bleeding and atrial arrhythmia also are risk factors of delayed extubation. In another study by London et al^23^ involving 304 patients undergoing cardiac surgery, age and inotrope use were found to be risk factors of delayed extubation. However, in their study extubation in ≤10 h was defined as early extubation. Arom et al^1^ in a retrospective review of CABG patients, wherein early extubation was defined as <12 h, reported that age, gender, preoperative diuretic use and presence of congestive heart failure or unstable angina were associated with delayed extubation. Cislaghi et al^24^ in a cohort study of 3,269 CABG patients demonstrated that redo surgery, longer CPB, intraoperative transfusion of more than 4 units of red blood cell or fresh frozen plasma, and LVEF of less than 30% are independent risk factors of delayed extubation, defined as needing mechanical ventilation longer than 12 h. Saleh et al^25^ in a retrospective study on 10,977 patients undergoing CABG showed that NYHA class of higher than II, renal dialysis, age, reduced FEV1, BMI of more than 35 kg/m^2^ are associated with increased risk of prolonged mechanical ventilation, with a cut-off point of 72 h for delayed extubation.

Advanced age reflects reduced physiological reserve and presence of co-morbid medical conditions. Contrary to our results, some previous studies have been recognized advanced age as the predictor of delayed extubation.^26-33^, However, our results demonstrated that there is no association between age and the risk of developing delayed extubation. Our results are in consistent with those of previous study by Branca et al^34^ which also found no association between BMI and delayed extubation. However, controversies exist in this regard and in some studies low BMI and in the others high BMI have been assumed as the risk factors of PMV.^29,35,36^

Our results revealed although most patients with the delayed extubation were female, but female gender was not associated with an increased risk of subsequent delayed extubation. This finding supports previous results.^16,26^ However, some other observational studies considered female gender as an independent risk factor of delayed extubation.^28,32,33^ Moreover, in consistent with previous report^33^, we found hypertension as an independent preoperative risk factor of delayed extubation. Similar to previous reports^26,28,30,32,33^, the presence of chronic renal failure could predict the occurrence of PMV in our study population. In parallel with some studies^28,32^ and in contrast with others^28,32^ we found a significant association between history of COPD and increased risk of prolonged mechanical ventilation. Similar to our study, Spivack et al^27^ defined delayed extubation as extubation of >48 h. They found a prevalence of approximately 8% to 9% of delayed extubation among patients undergoing CABG. They reported clinical heart failure as the risk factor of delayed extubation. However, their study demonstrated that none of history of COPD, bronchodilator use or spirometric function can be considered as risk factors of prolonged mechanical ventilation. Similar to previous studies^2,6,37^, in the present study no association was seen between history of hypothyroidism and delayed extubation.

We also found that neither LVEF of <30% nor hemoglobin concentration of <10 mg/dl can predict developing of delayed extubation. Moreover, Walthall et al^8^ believed that in patients with LVEF of less than 20% early extubation can be performed safely. However, in some other studies it has been shown that lower LVEF is associated with longer duration of mechanical ventilation.^11,38-41^, Gluck also showed that hemoglobin concentration cannot predict the occurrence of delayed extubation.^21^

Although it has been shown that patients with emergent cardiac surgeries have a higher risk of delayed extubation^11,15^ we demonstrated that timing of surgery does not affect the risk of prolonged mechanical ventilation. This may be due to the lower prevalence of emergent surgery in our study population (9% versus 91% of elective surgery).

In the present study, durations of surgery and CPB were statistically different in patients with early and delayed extubation. In multivariate analysis, in agreement with previous reports^11,42^, we also showed that prolonged duration of surgery and CPB increase the risk of delayed extubation.

In the present study we failed to find a predictive role of intraoperative transfusion for developing delayed extubation. However, study by Cislaghi et al^24^ revealed a significant association between intraoperative blood transfusion and increased risk of PMV.

Our results also revealed that bleeding during the first 24 h after surgery may be useful predictor of delayed extubation in patients undergoing open heart surgeries. This result supports previous reports by London et al^23^ which showed that postoperative excessive bleeding is an independent risk factor of prolonged mechanical ventilation. Moreover, similar to reports by London et al^23^, we showed that inotrope use during postoperation period is associated with increased risk of delayed extubation.

## Study limitations

The present study has some limitation which should be critically discussed. The main limitation of our study was its relatively small sample size, which might reduce generalizability of our results. In addition, we did not provid quantitative data regarding the number of units of packed cell transfusion and/or the volume of post-operative mediastinal bleeding. We also did not have sufficient data regarding the severity of hypertension in our patients. Moreover, data of the patients’ APACHE II score, which is indicative of the severity of disease in ICU admitted patients, was also lacking

## Conclusion

In summary, our study revealed that the history of hypertension, COPD, chronic kidney disease and previous endocarditis were the preoperative patient characteristics that can predict the occurrence of delayed extubation. Moreover, duration of surgery and CPB, bleeding and inotrope dependency during postoperative period could accurately predict delayed extubation after cardiac surgery. Our findings reinforce that risk stratification for predicting delayed extubation should be an important aspect of preoperative clinical evaluation in all anesthesiology settings.

## Ethical issues

The study was approval by the Local Ethics Committee.

## Competing interests

Authors declare no conflict of interests in this study.
